# The trend in delayed childbearing and its potential consequences on pregnancy outcomes: a single center 9-years retrospective cohort study in Hubei, China

**DOI:** 10.1186/s12884-022-04807-8

**Published:** 2022-06-24

**Authors:** Hui Li, Nawsherwan, Cuifang Fan, Sumaira Mubarik, Ghulam Nabi, Yin Xiao Ping

**Affiliations:** 1 Department of Medicine, Taixing People Hospital, Taizhou, Jiangsu China; 2 Department of Preventive Medicine, School of Health Sciences, Wuhan University, Wuhan, Hubei China; 3 Department of Obstetrics and Gynecology, Renmin Hospital, Wuhan University, Wuhan, Hubei China; 4 Department of Epidemiology and Biostatistics, School of Health Sciences, Wuhan University, Wuhan, Hubei China; 5 Institute of Nature Conservation, Polish Academy of Sciences, Krakow, Poland; 6 Department of Pediatrics, Taixing People Hospital, Taizhou, Jiangsu, China

**Keywords:** Delayed childbearing, Pregnancy complications, Adverse perinatal outcomes

## Abstract

**Background:**

Due to the advancement of modern societies, the proportion of women who delay childbearing until or beyond 30 years has dramatically increased in the last three decades and has been linked with adverse maternal-neonatal outcomes.

**Objective:**

To determine the trend in delayed childbearing and its negative impact on pregnancy outcomes.

**Material and methods:**

A tertiary hospital-based retrospective study was conducted in Wuhan University Renmin Hospital, Hubei Province, China, during the years 2011–2019. The joinpoint regression analysis was used to find a trend in the delayed childbearing and the multiple binary logistic regression model was used to estimate the association between maternal age and pregnancy outcomes.

**Results:**

Between 2011 and 2019, the trend in advanced maternal age (AMA ≥35 years) increased by 75% [AAPC 7.5% (95% CI: − 10.3, 28.9)]. Based on maternal education and occupation, trend in AMA increased by 130% [AAPC 11.8% (95% CI: 1.1, 23.7)] in women of higher education level, and 112.5% [AAPC 10.1% (95% CI: 9.4, 10.9)] in women of professional services. After adjusting for confounding factors, AMA was significantly associated with increased risk of gestational hypertension (aOR 1.5; 95% CI: 1.2, 2.1), preeclampsia (aOR 1.6; 95% CI: 1.4, 1.9), sever preeclampsia (aOR 1.7; 95% CI: 1.1, 2.6), placenta previa (aOR 1.8; 95% CI: 1.5, 2.2), gestational diabetes mellitus (aOR 2.5; 95% CI: 2.3, 2.9), preterm births (aOR 1.6; 95% CI: 1.4, 1.7), perinatal mortality (aOR 1.8; 95% CI: 1.3, 2.3), and low birth weight (aOR 1.3; 95% CI: 1.2, 1.4) compared with women aged < 30 years.

**Conclusion:**

Our findings show a marked increase in delayed childbearing and its negative association with pregnancy outcomes.

**Supplementary Information:**

The online version contains supplementary material available at 10.1186/s12884-022-04807-8.

## Introduction

Delayed childbearing or advanced maternal age (AMA) is defined as a mother of 35 years or older at the time of delivery. Due to the advancement of modern societies, availing of higher education, career development, and economic independence more young girls are likely to delay childbearing until or beyond 30 years of age [1–3]. This trend is more pronounced in industrialized countries, but rapidly becoming common in emerging countries. Maternal age at the time of first neonatal birth has dramatically increased in many countries. For example, in the United States, the childbearing age has increased from the early 20s in 1970 to later 20s in 2006. Similarly, in Switzerland women had their first child at nearly 30 years of age, five years older than Swiss women in 1970. In emerging countries, such as China, the childbearing age has increased from 24.3 years to 26.24 years during one decade (2000–2010) [4]. The birth rate in women of AMA has increased by 96.9% (from 8.65 to 17.04%) during 2004–2014, in China. On the other hand, the birth rate in women 25–29 years old decreased from 102.44 to 93.62% [5].

The AMA has been considered a significant risk factor associated with adverse pregnancy outcomes in both high and low-income countries [6]. Women of AMA are significantly associated with hypertensive disorders of pregnancy (HDP), gestational diabetes mellitus (GDM), placenta previa, preterm births, perinatal mortality, low birth weight (LBW), and congenital defects [5, 7–9]. Several studies have reported the impact of AMA on maternal-neonatal outcomes [5, 7–15]. However, the findings are conflicting. Some studies found an adverse impact of AMA on pregnancy outcomes [1, 8, 9, 16]. On the other hand, women of AMA had lower risk or were not significantly associated with adverse pregnancy outcomes [5, 11–13, 16]. An emerging third category even found positive outcomes, with an example of a recently published retrospective study from China which found a lower risk of adverse perinatal outcomes in women of AMA [5].

Women aged 30–34 years are also associated with an increased risk of adverse pregnancy outcomes. Many previous studies observed a significantly increased risk of adverse perinatal outcomes and pregnancy complications in women aged 30–34 compared with young mothers [1, 5, 8]. However, some prior studies have been neglected the maternal age group (30–34 years) during the classification of maternal age and its association with adverse pregnancy outcomes [7, 9, 14, 15]. Considering the continuously increasing trend in delayed childbearing and the previously reported conflicting findings of the association between AMA and adverse pregnancy outcomes. Therefore, we aimed to find the trend in delayed childbearing and verify the impact of AMA and maternal aged (30–34 years) on adverse pregnancy outcomes in Hubei, China.

## Material and methods

### Study population

A tertiary hospital-based retrospective study was conducted in the Wuhan University Renmin Hospital, Department of Obstetrics and Gynecology, Hubei, China from January 2011 to December 2019. The data was collected and documented in the obstetrics register and electronic database by trained nurses during individual examinations in the Gynecology and Obstetrics Department. The study protocol was approved by the Ethical Review Board of Renmin Hospital (ID: WDRY2019–K034) *in accordance with the Declaration of Helsinki.*

### Inclusion and exclusion criteria

**Fig. 1:**
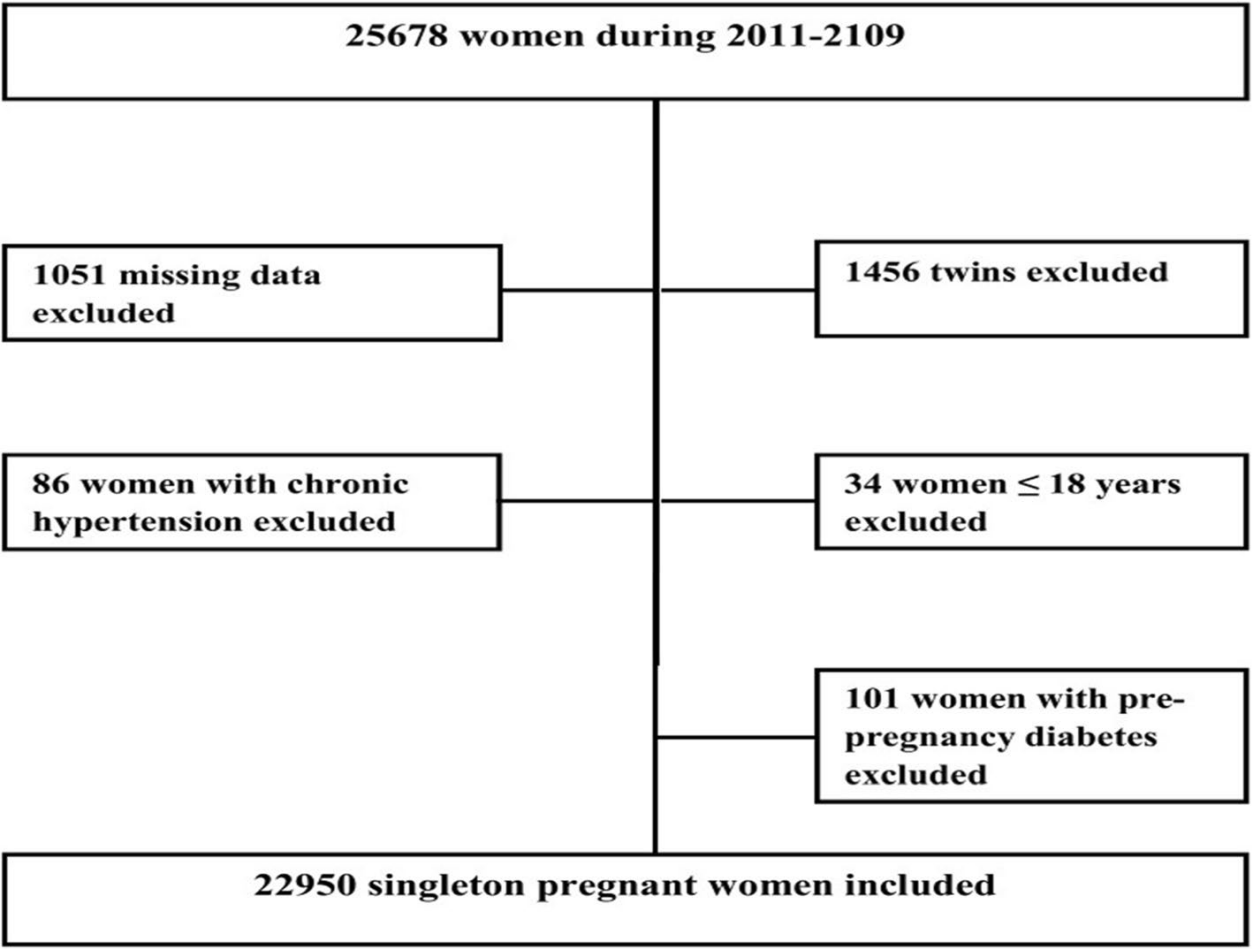
Flow chart of study population

A total of 23,051 singleton pregnant women were selected for the study. We excluded missing data on maternal age, pre-pregnancy body weight, neonatal gender, birth weight, birth length, and gestational age [17]. Pregnant women aged ≤18 years old, with chronic hypertension, pre-pregnancy diabetes, and twin neonates were also excluded from the analysis of data as shown in Fig. 1.

### Collection of data on maternal traits

Data regarding maternal traits were collected from the obstetrics register including maternal age, parity, prepregnancy body weight, gestational age, education, occupation, and pregnancy complications. At the time of delivery, based on age, pregnant women were divided into three groups (i) < 30 years, (ii) 30–34 years, (iii) and ≥ 35 years. Gestational age was calculated by the date of the last known menstrual period and confirmed by ultrasound examination during the first and second trimesters. Based on education, they were classified as ≤8 years (low), 9–12 years (middle), and ≥ 13 years (high). Maternal occupation categorized as (i) house-wives, (ii) professional services (doctors, nurses, accountants, teachers, lawyers, and actresses), and (iii) manual workers (farmers, waitresses, drivers, and factory workers).

### Definition of pregnancy complications and perinatal birth outcomes

Gestational hypertension (GH) is defined as having blood pressure greater than 140/90 mmHg without proteinuria after 20th weeks of gestation [18]. Preeclampsia (PE) is defined as elevated blood pressure 140/90 mmHg with proteinuria (albumin > 0.3 g in 24 hours) after the 20th week of gestation [19]. Sever PE referred to having a blood pressure higher than 160/110 mmHg with proteinuria (albumin > 5 g in 24 hours) after the 20th week of gestation [20]. Placenta previa is defined as suboptimal placental implantation near or over the cervical opening [21]. Placental abruption referred to the early separation of the placenta before childbirth [22]. Neonatal birth outcomes were recorded immediately after neonatal birth including birth weight in grams using an electronic infant scale, birth length in centimeters using a standard measuring board for the neonate. Preterm birth is defined as a neonate born before 37 completed weeks or fewer than 259 days from the first date of a woman’s last menstrual period [23]. Perinatal mortality is defined as the combination of late fetal mortality (stillbirths) and early neonatal mortality (0–6 days of life) [24]. Fetal macrosomia is defined as birth weight ≥ 4000 g and low birth weight (LBW) is defined as birth weight < 2500 g [25]. Intrauterine growth restriction (IUGR) is defined as a condition of fetal growth that is below the 10th percentile for its gestational age and does not reach its genetically predetermined growth potential [26]. Apgar score was determined by evaluating the newborn baby on five simple criteria on a scale from zero to two, then summing up the five values obtained. Apgar score was recorded at 1 minute, and at 5 minutes after birth. Apgar score was divided into two categories (i) low Apgar score (< 7), and (ii) normal Apgar score (≥ 7) [27]. Fetal hypoxia is defined as a pathophysiological condition in which the fetus is suffering from insufficient oxygen supply [28]. The ponderal index was determined by weight in kg / (length in m) ^3^ × 100. The ponderal index between 2.5 and 3.0 was considered normal, between 2.0 and 2.5 marginal, and a neonate with a ponderal index less than 2.0 was considered a low ponderal index (LPI) [29]. Congenital defect is defined as abnormality in the structure of neonatal body parts that occur during intrauterine development [30].

### Definition of confounding factors

Confounding factors were selected based on previous literature which are associated with both exposure and perinatal birth outcome [19]. The confounding factors included in this analysis were, pre-pregnancy body weight (≤ 45 kg and ≥ 91 kg), parity, and neonatal gender.

### Statistical analysis

Descriptive statistics such as frequencies and percentages were calculated for categorical and binary variables. A trend analysis using a chi-square test was conducted to compare baseline characteristics using groups of maternal age: Group 1 (< 30 years); Group 2 (30–34 years); Group 3 (≥35 years). Perinatal birth outcomes (i.e. preterm births, perinatal mortality, LBW, IUGR, LPI, low Apgar score, fetal hypoxia, macrosomia, and congenital defects) and pregnancy complications were considered as the outcome variables. Other variable such as the exposure variable (i.e. maternal age) was taken as a predictor variable. Multiple binary logistic regression models were used to find the association between maternal age and adverse pregnancy outcomes. The multiple binary logistic regression models were adjusted for confounding factors (maternal education, occupation, prepregnancy body weight ≤ 45 kg and ≥ 91 kg, parity, and neonatal gender). Adjusted odds ratios with 95% confidence intervals were used to estimate the association between predictor variables and outcome variables. *P-value* (two-tailed < 0.05) was taken as statistically significant. The data were analyzed by using SPSS (Statistical Package for Social Sciences) for window version 22 (IBM Corporation, Chicago, USA).

The trend in delaying childbearing was estimated by joinpoint regression analysis. In the regression analysis, for each segment/period, the annual percentage changes (APC) and the average annual percentage changes (AAPC) in the rate of delaying childbearing were determined. The AAPC represents the trend in delaying childbearing in the whole period 2011–2019; while, APC indicates the trend in delaying childbearing in each segment/period identified by the joint-point regression software. We presented the numbers of delaying childbearing change-points and estimated the model parameters by their associated *p-values* (< 0.05). Moreover, Monte Carlo methods were used to find each *p-value* and maintain the overall asymptotic significance level through Bonferroni correction. This analysis was conducted using the Join-point regression program version 4.8.0.1 (April 2020) from the Surveillance Research Program of the U.S. National Cancer Institute.

## Results

### General characteristics of pregnant women and neonates

Among these studied women (*n* = 22,950), 82.8% were younger than 35 years of age and 17.2% were equal or older than 35 years of age. Compared to women of age group < 30 years, women of AMA had a significantly higher prevalence of hypertensive disorders of pregnancy, abnormal placentation, cesarean section, gestational diabetes mellitus (GDM), preterm births, perinatal mortality, LBW, low Apgar score, and macrosomic babies (*p* < 0.05) (Tables 1 and 2).

**Table 1:** Distribution of maternal traits and pregnancy complications by maternal age groups (*N* = 22,950)

Maternal traits, and pregnancy complications	Groups of maternal age			
	G1 (*n* = 11,282) < 30 years No. %	G2 (*n* = 7732) 30–34 years No. %	G3 (*n* = 3936) ≥35 years No. %	*P*-value
Maternal education				
Low	2458 (21.8)	1494 (19.3)	965 (24.6)	0.001
Middle	4575 (40.6)	2947 (38.1)	1517 (38.5)	
Higher	4249 (37.6)	3291 (42.6)	1454 (36.9)	
Maternal occupation				
Housewives	6046 (53.6)	3810 (49.3)	2135 (54.3)	0.001
Professional services	4964 (44)	3751 (48.5)	1702 (43.2)	
Manual workers	272 (2.4)	171 (2.2)	99 (2.5)	
Parity				
Primiparous (≤1)	9494 (84.2)	5606 (72.5)	2302 (58.5)	0.001
Multiparous (> 1)	1788 (15.8)	2126 (27.5)	1634 (41.5)	
Cesarean section^*^	5993 (53.1)	5032 (65.1)	2905 (73.8)	0.001
Previous history of cesarean section ^*^	991 (8.8)	1484 (19.2)	1103 (28.0)	0.001
HDP				
GH^*^	119 (1.1)	96 (1.2)	64 (1.6)	0.02
PE^*^	451 (4.0)	398 (5.1)	261 (6.6)	0.001
Sever PE^*^	63 (0.6)	36 (0.5)	34 (0.9)	0.02
Abnormal Placentation				
Placenta previa^*^	384 (3.4)	331 (4.3)	247 (6.3)	0.001
Placental abruption^*^	24 (0.2)	16 (0.2)	12 (0.3)	0.5
Others pregnancy complications				
PROM^*^	1126 (10.0)	681 (8.8)	339 (8.6)	0.005
GDM^*^	517 (4.6)	587 (7.6)	429 (10.9)	0.001
Fetal breech presentation^*^	267 (2.4)	195 (2.5)	109 (2.8)	0.3
Oligohydramnios^*^	411 (3.6)	281 (3.6)	108 (2.7)	0.02
Polyhydramnios^*^	51 (0.5)	28 (0.4)	11 (0.3)	0.3
Nuchal cord^*^	533 (4.7)	309 (4.0)	150 (3.8)	0.01

* = Frequency and percentage of variables with only ‘Yes’ value presented**,** *HDP *Hypertensive disorders of pregnancy, *GH *Gestational hypertension, *PE *Preeclampsia, *PROM *Premature rupture of membrane, *GDM *Gestational diabetes mellitus, *p-values* were calculated using chi-square test

**Table 2:** Distribution of perinatal traits by maternal age groups (*N* = 22,950)

Perinatal traits	Groups of maternal age			
	G1 (*n* = 11,282) < 30 years No. %	G2 (*n* = 7732) 30–34 years No. %	G3 (*n* = 3936) ≥35 years No. %	*P*-value
Preterm birth^*^	2003 (17.8)	1376 (17.8)	1009 (25.6)	0.001
Perinatal mortality^*^	147 (1.3)	86 (1.1)	89 (2.3)	0.001
LBW^*^	1575 (14.0)	985 (12.7)	693 (17.6)	0.001
IUGR^*^	88 (0.8)	56 (0.7)	23 (0.6)	0.4
LPI^*^	434 (3.8)	293 (3.8)	165 (4.2)	0.5
Low Apgar score^*^	403 (3.6)	247 (3.2)	181 (4.6)	0.001
Fetal hypoxia^*^	255 (2.3)	176 (2.3)	88 (2.2)	0.9
Macrosomia^*^	555 (4.9)	473 (6.1)	212 (5.4)	0.002
Congenital defects*^a^	157 (1.4)	85 (1.1)	53 (1.3)	0.1
Neonatal gender				
Male	5975 (53)	4133 (53.5)	2170 (55.1)	0.06
Female	5307 (47)	3599 (46.5)	1766 (44.9)	

* = Frequency and percentage of only ‘Yes’ value presented**,*** LBW* Low birth weight, *IUGR *Intrauterine growth restriction, *LPI *Low ponderal index, low Apgar score (< 7), fetal hypoxia (a pathophysiological condition in which the fetus is suffering from insufficient oxygen supply), ^a^Congenital defects (microtia, anotia, polydactyly, heart defects, limb reduction defects, cleft lip, cleft palate, hydrocephaly, and NTDs), *p-values* were calculated using chi-square test

### Temporal trends of maternal age groups in different time segments identified by the joinpoint regression analysis

**Fig. 2:**
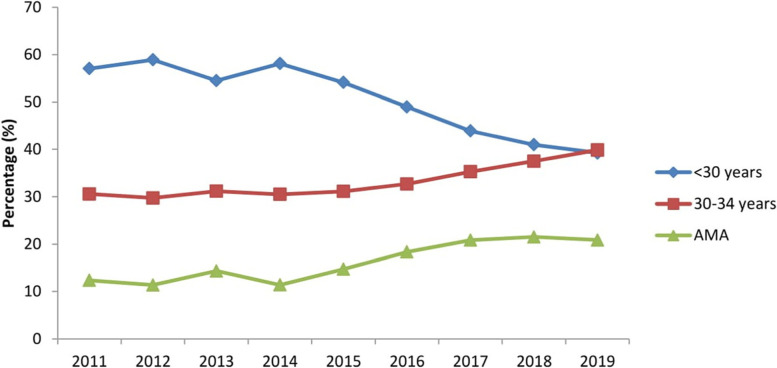
Trend of maternal age groups accros different years of study (2011–2019)

The joinpoint regression analysis revealed that trend of maternal age groups (30–34 years) and AMA increased by 29.1% [AAPC 3.4% (95% CI: − 0.3, 7.2)] and 75% [AAPC 7.5% (95% CI: − 10.3, 28.9)], respectively from 2011 to 2019 (Table 3 & Fig. 2).

**Table 3:** Trends of maternal age groups in pregnant women using joinpoint regression analysis from 2011 to 2019

Variables and segments	Year	APC (95% CI)
< 30 years		
Trend1	2011–2014	0.2 (−36.2, 57.5)
Trend2	2014–2017	−8.2 (− 62.8, 126.4)
Trend3	2017–2019	−6.6 (− 62.1, 130.3)
AAPC (95% CI)	2011–2019	−4.7 (−11.0, 2.0)
30–34 years		
Trend1	2011–2014	−1.0 (−22.2, 25.9)
Trend2	2014–2017	5.3 (−34.9, 70.3)
Trend3	2017–2019	7.4 (−33.6, 73.7)
AAPC (95% CI)	2011–2019	3.4 (−0.3, 7.2)
≥35 years (AMA)		
Trend1	2011–2014	0.4 (− 69.9, 34.5)
Trend2	2014–2017	21.4 (− 89.1, 248.5)
Trend3	2017–2019	−0.8 (−91.1102.0)
AAPC (95% CI)	2011–2019	7.5 (−10.3, 28.9)

*APC* Annual percentage change, *AAPC A*verage annual percent change, *CI C*onfidence interval

### Temporal trends of AMA in pregnant women based on education and occupation in different time segments identified by the joinpoint regression analysis

Based on education, the trend of AMA increased in pregnant women of middle education by 11.1% [AAPC 1.5% (95% CI: − 4.0, 7.3)] and in women of higher education by 130% [AAPC 11.8% (95% CI: 1.1, 23.7)] from 2011 to 2019. Moreover, the trend of AMA in pregnant women of professional services increased by 112.5% [AAPC 10.1% (95% CI: 9.4, 10.9)] during the study period (Table S1, Fig. S1 & S2).

### Association of maternal age with pregnancy complications

We observed that AMA was an independent and statistically significant risk factor associated with increased risk of GH (aOR 1.5; 95% CI: 1.2, 2.1), PE (aOR 1.6; 95% CI: 1.4, 1.9), sever PE (aOR 1.7; 95% CI: 1.1, 2.6), placenta previa (aOR 1.8; 95% CI: 1.5, 2.2), GDM (aOR 2.5; 95% CI: 2.3, 2.9), and cesarean section (aOR 2.4; 95% CI: 2.2, 2.6). Women of 30–34 years aged had higher odds of PE (aOR 1.3; 95% CI: 1.2, 1.5), placenta previa (aOR 1.2; 95% CI: 1.1, 1.4), GDM (aOR 1.7; 95% CI: 1.5, 1.9), and cesarean section (aOR 1.6; 95% CI: 1.5, 1.7) (Table 4).

**Table 4:** Association of maternal age with hypertensive disorders of pregnancy, abnormal placentation, and others pregnancy complications

Pregnancy complications	Maternal age groups		
	G1 (< 30 years) aOR (95% CI)	G2 (30–34 years) aOR (95% CI)	G3 (≥35 years) aOR (95% CI)
HDP			
GH	1.00 (reference)	1.2 (0.9, 1.6)	1.5 (1.2, 2.1)
PE	1.00 (reference)	1.3 (1.2, 1.5)	1.6 (1.4, 1.9)
Sever PE	1.00 (reference)	0.8 (0.5, 1.3)	1.7 (1.1, 2.6)
Abnormal Placentation			
Placenta previa	1.00 (reference)	1.2 (1.1, 1.4)	1.8 (1.5, 2.2)
Placental abruption	1.00 (reference)	0.9 (0.5, 1.8)	1.4 (0.7, 2.8)
Cesarean section	1.00 (reference)	1.6 (1.5, 1.7)	2.4 (2.2, 2.6)
Others pregnancy complications			
PROM	1.00 (reference)	0.8 (0.7, 0.9)	0.8 (0.7, 1.0)
GDM	1.00 (reference)	1.7 (1.5, 1.9)	2.5 (2.3, 2.9)
Fetal breech presentation	1.00 (reference)	1.1 (0.9, 1.3)	1.2 (0.9, 1.4)
Oligohydramnios	1.00 (reference)	0.9 (0.8, 1.2)	0.7 (0.6, 1.1)
Polyhydramnios	1.00 (reference)	0.8 (0.5, 1.2)	0.6 (0.3, 1.1)
Nuchal cord	1.00 (reference)	0.8 (0.7, 0.9)	0.8 (0.6, 0.9)

*aOR *Adjusted odds ratios, *HDB *Hypertensive disorders of pregnancy, *GH *Gestational hypertension, *PE *Preeclampsia, *PROM *Premature rupture of membrane, *GDM* Gestational diabetes mellitus, Adjusted for prepregnancy body weight, parity, neonatal gender, maternal education, and occupation;

### Association of maternal age with adverse perinatal outcomes

Compared with maternal age group < 30 years, women of AMA had higher odds of preterm births (aOR 1.6; 95% CI: 1.4, 1.7), perinatal mortality (aOR 1.8; 95% CI: 1.3, 2.3), and LBW (aOR 1.3; 95% CI: 1.2, 1.4). Women of age group 30–34 years had an increased risk of macrosomia (aOR 1.2; 95% CI: 1.1, 1.4) (Table 5).

**Table 5:** Association of maternal age with adverse perinatal outcomes

Perinatal traits	Maternal age groups		
	G1 (< 30 years) aOR (95% CI)	G2 (30–34 years) aOR (95% CI)	G3 (≥35 years) aOR (95% CI)
Preterm birth	1.00 (reference)	1.1 (0.9, 1.2)	1.6 (1.4, 1.7)
Perinatal mortality	1.00 (reference)	0.8 (0.6, 1.1)	1.8 (1.3, 2.3)
LBW	1.00 (reference)	0.9 (0.8, 1.1)	1.3 (1.2, 1.4)
IUGR	1.00 (reference)	0.9 (0.6, 1.2)	0.7 (0.4, 1.1)
LPI	1.00 (reference)	1.0 (0.8, 1.2)	1.1 (0.9, 1.3)
Low Apgar score	1.00 (reference)	0.9 (0.8, 1.1)	1.3 (1.1, 1.6)
Fetal hypoxia	1.00 (reference)	1.1 (0.8, 1.3)	1.1 (0.8, 1.4)
Macrosomia	1.00 (reference)	1.2 (1.1, 1.4)	1.1 (0.9, 1.3)
Congenital defects	1.00 (reference)	0.8 (0.6, 1.1)	0.9 (0.7, 1.3)

*aOR *Adjusted odds ratios, *LBW *Low birth weight, *IUGR *Intrauterine growth restriction, *LPI *Low ponderal index, Adjusted for prepregnancy body weight, parity, neonatal gender, maternal education, and occupation;

## Discussion

In the present tertiary hospital-based retrospective study (2011–2019), we observed an increasing trend in delayed childbearing and its adverse effect on the hypertensive disorder of pregnancy, abnormal placentation, GDM, and perinatal outcomes.

### Trend in delayed childbearing or AMA in the study period (2011–2019)

Our findings revealed increasing trends in the AMA. Our results are consistent with several studies that reported an increasing trend in the Chinese population during different periods. For example, in a national based-hospital surveillance study, Li et al. [31] found that trend of AMA increased from 2.96 to 8.56% during 1996–2007. In urban areas, the trend of AMA increased from 2.95 to 7.69% while, in rural areas, the trend of AMA increased from 2.99% in 1996 to 10.35% in 2007. The trend of AMA was higher in rural areas than in urban areas. In our study, the trend of AMA increased by 75% (from 12 to 21%) with an AAPC of 7.5% during 2011–2019. Our data represents the trend of AMA in pregnant women of urban areas however, due to lack of data; we couldn’t find the trend of AMA in rural areas.

In Zhejiang province, the number of women with AMA increased from 8.83 to 10.08% during 2011–2015 [32]. The proportion of neonatal births to women with AMA increased by 85.68% from 8.52% in 2013 to 15.82% in 2017 [33]. AMA is a well-known risk factor associated with pregnancy complications and adverse perinatal outcomes, therefore the increasing trend of AMA in the Chinese population is of great concern and should pay serious attention.

We observed that trend of AMA increased in women with higher education levels and women of professional services. Women who aspire to achieve higher educational levels are more likely to delay childbearing [34]. Furthermore, women with higher educational degrees are likely to pursue their careers, which may postpone childbearing until they are well established on their career path [35]. Montilva [36] explored that the development of professional career and academic training are the reasons that can delay childbearing in the South American population. Many women of professional jobs planned to have kids at later age to protect and promote their careers [4].

Although women of professional occupation get benefits to delay childbearing such as pursuing higher levels of education and career development. However, delayed childbearing is associated with both maternal pregnancy complications and increased risk of adverse perinatal outcomes Therefore, it is essential to inform women of professional occupation that delayed childbearing could potentially affect conception, pregnancy, and perinatal outcomes.

### AMA and pregnancy complications

Our findings depict that women of AMA had an increased risk of hypertensive disorders of pregnancy (HDP). AMA is recognized as an independent risk factor for HDP [37, 38]. In a retrospective cohort study, AMA was associated with an increased risk of HDP [7]. The association between AMA and increased risk of HDP is not enough clear. However, low nitric oxide levels and high oxidative stress are signs of aging, which could adversely affect the relaxation of the endothelium. This may cause the development of pregnancy-induced hypertension (PIH) in women with AMA because pregnancy increases cardiac output [39].

AMA upsurges the risk of placenta previa and GDM. We found that women with AMA are associated with increased risk of placenta previa and GDM. The increased risk of placenta previa among older women could be attributed to atherosclerotic changes in the uterine blood vessels that cause compromised uteroplacental blood flow [40]. Moreover, women with AMA had a higher incidence of multiparity and previous Cesarean section history in our study. Multiparty and previous Cesarean section history could also increase the risk of placenta previa in older women [41].

Several previous studies have confirmed a strong positive association between AMA and GDM development [42, 43] and even after adjusting for confounding factors [43]. The association between AMA and GDM development could be explained by the progressive vascular endothelial damage in women of older ages [44]. Fulop et al. [45] reported a reduction in insulin sensitivity, impaired glucose tolerance, and deterioration of pancreatic β-cell function [46] as maternal age increases.

### AMA and adverse perinatal outcomes

We found that women with AMA had a higher risk of preterm births, perinatal mortality, and LBW compared with women aged < 30 years. Adverse perinatal outcomes, including preterm births, perinatal mortality, and LBW, are most common among neonates born to mothers with advanced age (≥35 years) [47–49]. The underlying causes between AMA and preterm births are still not clear. However, placental vascular pathology [50] and progesterone deficiency in women of AMA may associate with preterm births [51].

Many studies reported that AMA is associated with an increased risk of perinatal mortality [52, 53]. The biological mechanisms associated with increased risk of perinatal mortality and AMA remain elusive [54]. Myometrial hypo-perfusion due to sclerotic arterial lesions [55] and the aging endothelium of women of AMA are considered to be associated with perinatal complications. Women of AMA are often associated with PIH and GDM [48] and more than half of pregnant women experienced stillbirths having pregnancy complications [56].

Maternal age at delivery is known to be associated with neonatal birth weight. Women aged ≥35 years are at higher risk to have LBW neonates compared with women aged 20–34 years [57, 58]. In addition to AMA, several risk factors were associated with increased risk of LBW in the previously published studies. However, in our study women with AMA had higher odds of GH, PE, severe PE, placenta previa, and GDM. These pregnancy complications in women with AMA could attribute to an increase in the risk of LBW in our study.

### Maternal age (30–34 years) and adverse pregnancy outcomes

In our findings**,** women aged 30–34 years had higher odds of PE, placenta previa, GDM, diabetes, fetal macrosomia, and Cesarean section compared with women aged < 30 years. These findings are consistent with the previously published reports describing the adverse impact of maternal age on pregnancy outcomes. For example, Waldenstrom et al. [1] observed a significantly increased risk of very preterm births, SGA, and neonatal death in women aged 30–34 compared with 25–29 year old women in the Sweden population. Similarly, several studies reported an increased risk of adverse pregnancy outcomes in women aged 30–34 years [5, 8].

In another population-based register study, Waldenstrom et al. [59] found an increased risk of preterm births in women aged (30–34 years). However, Almeida et al. [60] and Fuchs et al. [61] reported no association between women aged (30–34 years) and adverse pregnancy outcomes. These differences in findings could be related to the different definitions of AMA. For investigating the effects of maternal aging on adverse pregnancy outcomes, the cutoff value for age groups and the definition of the reference group is very crucial. If, for example, women of ≥35 years old are compared with women less than 35 years of age, the effect of maternal aging could be underestimated because of the U-shaped distribution of the adverse pregnancy outcomes [1].

Our findings depicted that women aged (30–34 years) were also significantly associated with an increased risk of fetal macrosomia. In this study, women aged (30–34 years) had comparatively higher prevalence of GDM compared with the reference group. Several previous studies had reported the association between GDM, diabetes mellitus, and fetal macrosomia [62–64]. It suggests that fetal macrosomia in women aged (30–34 years) may be because of a higher prevalence of GDM.

## Limitations

We acknowledge that our study had certain limitations. The study design was retrospective. Our data analysis is based on a single center tertiary hospital, which is the potential selection bias in this study. The study was lack of collected information related to assisted reproductive technology, maternal’s obesity, smoking, and drinking habits. In addition, the impact of attributable risk factors (i.e. maternal education and occupation) of delayed childbearing has not been investigated on pregnancy outcomes. We had a low sample size and the results cannot be generalized to the whole population.

## Conclusion

In conclusion, the trend of delayed childbearing increased in women during 2011–2019. Moreover, regardless of the underlying mechanism, our results confirm the negative impact of AMA and maternal aged (30–34 years) on pregnancy outcomes. These findings should be carefully taken into account by maternal health caregivers to educate women about the consequences of delayed childbearing and provide evidence-based knowledge to support women about their procreation choices. Moreover, women aged 30–34 years could not be ignored during categorizing maternal age into groups.

## Supplementary Information

**Additional file 1.**

## Data Availability

All data analyzed during this study are included in this article.
