# Supplementary material for: The trend in delayed childbearing and its potential consequences on pregnancy outcomes: a single center 9-years retrospective cohort study in Hubei, China
Source: BMC Pregnancy Childbirth. 2022 Jun 24;22:514. doi: 10.1186/s12884-022-04807-8 (PMC9233367; doi:10.1186/s12884-022-04807-8)
Supplement: Supplementary file 1 — Additional file 1. [file 12884_2022_4807_MOESM1_ESM.docx]

**Table S1:** Trends of advanced maternal age (AMA) in pregnant women based on education and occupation using joinpoint regression analysis from 2011-2019.

| Variables and segments | Year | APC (95% CI) |
| --- | --- | --- |
| Lower education |  |  |
| Trend1 | 2011-2014 | -8.5 (-48.1, 61.3) |
| Trend2 | 2014-2017 | -16.3 (-73.1, 160.4) |
| Trend3 | 2017-2019 | -15.4 (-72.8, 163.1) |
| AAPC (95% CI) | 2011-2019 | -13.2 (-20.3, -5.5) |
| Middle education |  |  |
| Trend1 | 2011-2013 | 2.3 (-51.1, 114.1) |
| Trend2 | 2013-2016 | -1.2 (-52.8, 106.8) |
| Trend3 | 2016-2019 | 3.7 (-28.3, 50.1) |
| AAPC (95% CI) | 2011-2019 | 1.5 (-4.0, 7.3) |
| Higher education |  |  |
| Trend1 | 2011-2014 | 17.9 (-39.6, 130.1) |
| Trend2 | 2014-2017 | 11.6 (-70.7, 325.2) |
| Trend3 | 2017-2019 | 3.6 (-72.8, 294.5) |
| AAPC (95% CI) | 2011-2019 | 11.8 (1.1, 23.7) |
| Housewives |  |  |
| Trend1 | 2011-2013 | -4.9 (-40.2, 51.1) |
| Trend2 | 2013-2016 | -7.9 (-42.1, 46.4) |
| Trend3 | 2016-2019 | -3.8 (-23.7, 21.3) |
| AAPC (95% CI) | 2011-2019 | -5.6 (-8.9, -2.3) |
| Professional job holders |  |  |
| Trend1 | 2011-2014 | 16.4 (11.2, 21.8) |
| Trend2 | 2014-2017 | 9.5 (4.5, 19.9) |
| Trend3 | 2017-2019 | 2.3 (-6.7, 12.0) |
| AAPC (95% CI) | 2011-2019 | 10.1 (9.4, 10.9) |
| Manual workers |  |  |
| Trend1 | 2011-2014 | -20.5 (-95.4, 179.5) |
| Trend2 | 2014-2017 | 21.6 (9.6, 87.8) |
| Trend3 | 2017-2019 | -6.0 (-99.7, 85.1) |
| AAPC (95% CI) | 2011-2019 | -2.7 (-36.7, 49.5) |

**Note:** APC (annual percentage change), APPC (average annual percent change), CI (confidence interval)
